# Genome-wide association study reveals key genomic regions underlying colostrability in Italian Holstein cattle

**DOI:** 10.1186/s12711-026-01080-7

**Published:** 2026-08-20

**Authors:** Angela Costa, Massimo De Marchi, Jacopo Vegni, Arianna Goi, Silvia Magro, Martino Cassandro, Raffaella Finocchiaro, Maurizio Marusi, Lorenzo Benzoni, Jan-Thijs van Kaam, Giovanni Niero, Giulio Visentin

**Affiliations:** 1 Department of Veterinary Medical Sciences, Alma Mater Studiorum University of Bologna, Ozzano dell’Emilia, BO Italy; 2 Department of Agronomy, Food, Natural Resources, Animals and Environment, University of Padova, Legnaro, PD Italy; 3 Associazione Nazionale Allevatori della Razza Frisona, Bruna e Jersey Italiana (ANAFIBJ), Cremona, Italy

## Abstract

**Background:**

Colostrum yield (CY, L) and concentration of immunoglobulin G (IgG, g/L) are important phenotypes to monitor in dairy farms because of their association with the risk of failure of the passive transfer of immunity in the newborn calf. This can occur when the CY of the parturient cow is insufficient or when the IgG concentration is low. Given that both of these traits are heritable, the present study aimed to investigate their genetic determinism by identifying significant genomic regions. A genome-wide association study coupled with an exploratory functional enrichment analysis was carried out to provide preliminary biological context of the detected signals for the ‘colostrability’ defined as the cow’s ability to secrete enough volume (≥ 4 L) of good-quality colostrum (≥ 50 g IgG/L) at calving. Data comprised 960 genotyped Italian Holstein cows with CY recorded within 6 h of calving, together with colostral IgG and total immunoglobulin concentrations.

**Results:**

The significant SNPs associated with CY were scattered across BTA3, 5, 10, 11, 21, and 22, with 23 genes on BTA22 either harbouring or flanking significant signals. Apart from some genes already known, part of the significant regions have unclear function. Signals were detected on BTA1, 6, 7, 11, 19, 21, and 25 for IgG concentration, and on BTA6, 7, 11, 21, 23, and 25 for total immunoglobulin concentration. The functional enrichment analysis provided preliminary support for possible involvement of secretory, immune-signalling, and epithelial receptor-related processes.

**Conclusions:**

This study confirms the polygenic nature of cows’ ‘colostrability’ being regulated by different genomic regions distributed across the genome. However, exploration of the genomic determinism of CY and immunoglobulin concentration requires larger, independent, and harmonized data, ideally standardized and highly comparable. These findings, although relevant for improving calf health, represent only part of a more complex picture when the goal is selective breeding toward calf health. In addition to dam-related data, including colostrum traits, future studies should integrate calf-related phenotypes associated with failure of passive transfer of immunity, such as intestinal IgG absorption capacity, gut permeability, early-life survival, and health outcomes.

**Supplementary Information:**

The online version contains supplementary material available at 10.1186/s12711-026-01080-7.

## Background

We define here the concept of colostrability, the cow’s ability to secrete enough volume (≥ 4 L) of good-quality colostrum at calving in due time. Indeed, the passive transmission of antibodies, especially immunoglobulin G (IgG), from the dam to the newborn through colostrum provided at the first meal is pivotal because of the agammaglobulinemic status at birth [1–4]. Therefore, timely administration is fundamental for neonatal calf survival. Conventionally, for farmers, operators, and veterinarians, the concept of quality relies on the colostrum concentration of IgG [1], which should be at least 50 g/L in cattle to reduce the risk of failure of the passive transfer of immunity [5–7]. Failure occurs when the calf serum level of IgG does not reach 12 g/L in the first 12 h of life. Like many other complex phenotypes, the volume of colostrum produced at first milking (CY, L) is significantly affected by parity, season, vaccination, and overall farm management and only partly determined by genetics, with generally low heritability (h^2^) in various breeds [8–10]. Similarly, limited genetic variability has been reported for colostral solid concentration measured via refractometers, an indirect proxy of quality [9, 10]. Although large-scale CY and IgG data collection may be considered infeasible because time-consuming and costly, recent efforts have been made to increase phenotypes recording and investigate colostrum traits’ variability. Since mammary gland secretion in the first 4 days of lactation is not intended for the bulk tank, there is usually no precise quantification of CY at the first milking and transition milk, which are thereby commonly overlooked and not recorded, making the evaluation of parturient cows’ superiority difficult to implement. In the same manner, colostrum traits are extremely difficult to record on farms where dams and calves are kept together, with consequently no access to the real values of CY and IgG concentration. For these reasons, genomic aspects remain still poorly investigated [5], and genes and pathways involved are not well understood yet. In addition to advancing basic biological knowledge, findings from genetic studies may have practical applications in breeding, enabling marker-assisted and selective breeding. Therefore, evaluating cows’ genomic predisposition to colostrability through the identification of key genomic regions represents an initial step toward exploring the possibility of developing polygenic risk scores. In the present study, phenotypes and SNP data of Italian Holstein cows were used to carry out a genome-wide association study for CY, IgG concentration, total Ig (IgTOT) concentration, and IgG yield at calving.

## Methods

### Phenotypes

The data belonged to 960 genotyped cows and consisted of CY at first milking, and IgG and IgTOT concentrations determined with existing near-infrared spectroscopy prediction models [11], whose coefficients of determination in external validation were 0.82 for the concentration of IgG and 0.84 for the concentration of IgTOT. The sampling procedures and protocol provided to the 31 farmers were described by Costa et al. [8, 12]. According to it, in this study CY referred exclusively to the first colostrum milked within 6 h from calving with total removal (empty milking) and was measured using a graduated bucket provided to the farmers in advance. Therefore, the timely separation of calf from the dam was always ensured to avoid biasing phenotypic data [12]. For the same reason, data related to unsupervised calvings were excluded from the study.

After collection in plastic sterile tubes, colostrum samples were frozen by the farmers after registration and labelling to be stored until processing at the laboratory for IgG concentration assessment via near-infrared spectroscopy [11].

By multiplying the CY by the IgG concentration, IgG yield (g) was calculated and used as an additional phenotype for the association study. This trait was included as a composite indicator of colostrability, as it represents the amount of IgG potentially available and absorbable by the calf at the first meal.

After editing, data from 296, 232, 174, 109, and 101 cows in parity 1, 2, 3, 4, and 5 were available, respectively.

### Genotypes

The number of genotyped cows per farm ranged from 1 to 147. Because SNP array density differed among genotyping platforms, ANAFIBJ provided imputed genomic data (86,339 markers across BTA1–29) obtained with the PedImpute software [13]. Overall, the number of genotyped SNPs across the 56 chips averaged 71,628, with minimum and maximum of 2900 and 777,962, respectively. For the 960 cows with phenotypes, the quality control was performed with PLINK 2.0 [14] by considering a maximum for missing rate for both SNPs (10%) and cows (> 10%), Hardy–Weinberg equilibrium (*P* > 0.000001), and minor allele frequency (0.05). Quality control resulted in 69,806 markers across the autosomes which were used in a principal component analysis to detect possible clusters arising from population structure.

### GWAS

The following linear mixed model was adopted in the GCTA software v.1.93.3 [15] for the association analysis:

1 $${\mathbf{y}} = {\mathbf{1}}\upmu + {\mathbf{x}}\upbeta + {\mathbf{H\gamma }} + {\mathbf{P\delta }} + {\mathbf{u}} + {{\boldsymbol{\upvarepsilon}}},$$

where **y** is the vector of the phenotype (CY, IgG concentration, IgTOT concentration, or IgG yield); μ is the intercept of the model; **1** is a vector of ones; **x** is a vector of SNP genotypes with β being the effect size; **H** and **P** are incidence matrices for the fixed effects herd-season (n = 77; June to August for summer, September to November for autumn, December to February for winter, and March to May for spring) and parity, respectively, with **γ** and **δ** being vectors of corresponding estimated effects; **u** is a vector of random individual effects; and **ε** is the vector of residual errors. The herd-season effect was included to account for differences due to variable management and environmental conditions such as within-herd changes in housing, feeding, and caring potentially occurring during the year. Log-likelihood ratio statistics were adopted to test the null hypothesis that a polymorphism did not affect the phenotype. The genomic relationship matrix built in GCTA using the VanRaden method [16] was included in the analysis to account for potential population structure. Since the Bonferroni correction ignores that some SNPs trace the same genomic region because of linkage disequilibrium between loci [17], the false discovery rate (FDR) with a cut-off of 0.20 (*P* < 0.00013) was used for significance attribution [18]. This less stringent threshold aligns with the exploratory nature of the study which is intended to identify genomic regions deserving future attention. After the logarithmic transformation of *P*-values, Manhattan and quantile–quantile plots were obtained adopting the packages ‘CMplot’ [19] and ‘qqman’ packages [20] available in R software version 4.3.2 [21].

### Gene annotation and functional enrichment

Regions containing significant signals were selected, to detect coding regions in which the SNP fell (± 0.5 Mb), using the ENSEMBL Biomart Martview application (Ensembl Genes 97, http://www.ensembl.org/biomart/martview). The reference *Bos Taurus* genome assembly ARS-UCD 1.3 was adopted for gene identification and subsequent functional enrichment analysis, which was performed with DAVID (https://davidbioinformatics.nih.gov) [22]. The gene ontology (GO) terms, which included molecular function (MF), cellular component (CC), biological process (BP), and Kyoto Encyclopedia of Genes and Genomes (KEGG) pathways with *P* ≤ 0.05 were extrapolated for GO analysis. The results obtained from the functional enrichment analysis, used as an exploratory tool for biological contextualization, were subsequently used to produce bubble plots to illustrate the most relevant functional categories and a Venn diagram was generated for visual representation of overlapping significant regions/loci [23].

## Results

### Data overview

The descriptive statistics of the traits investigated are reported in Table 1 and the data distributions are available as supplementary material [Additional file 1, Figure S1]. On average, cows yielded 4.55 L of colostrum at first milking, and some of them produced very low quantities (0.12 L). Overall, CY was moderately inversely correlated with the two concentration traits (Table 1). The percentage of cases where CY at the first milking was insufficient (< 4 L) was the greatest in parity 1 (87%; 138 cows). For parity 2, 3, 4, and 5 this percentage was 37%, 50%, 49%, and 55% respectively. In addition, regardless of parity, autumn was the season with the lowest CY, with 40% of cows delivering insufficient amount of colostrum. In 10 farms the average CY was < 4 L, while the CY averaged 8.58 L in the top 3 farms. In terms of IgG, the lowest average concentration was observed in cows that calved in spring (87.04 g/L) and in first-parity cows (89.87 g/L). In contrast, autumn-calving cows (107.45 g/L) and those in parity 5 (124.55 g/L) had the highest IgG concentration. The average CY, IgG, IgTOT and IgG yield for each herd are reported in the supplementary table S1 [Additional file 2, Table S1].

**Table 1:** Descriptive statistics of the colostrum traits investigated and their Pearson correlations (*P* < 0.001)

Trait	Mean	SD	Minimum	Maximum	IgG	IgTOT	IgG yield
CY, L	4.55	2.45	0.12	24.00	− 0.221	− 0.223	0.783
IgG, g/L	101.42	33.75	13.26	218.90		0.987	0.342
IgTOT, g/L	114.31	37.26	19.61	249.95			0.327
IgG yield, g	442.91	243.58	12.66	2272.56			–

CY = colostrum yield (L); IgG = immunoglobulin G concentration (g/L); IgTOT = total immunoglobulin concentration (g/L); IgG yield = immunoglobulin G yield (g)

### GWAS

**Fig. 1:**
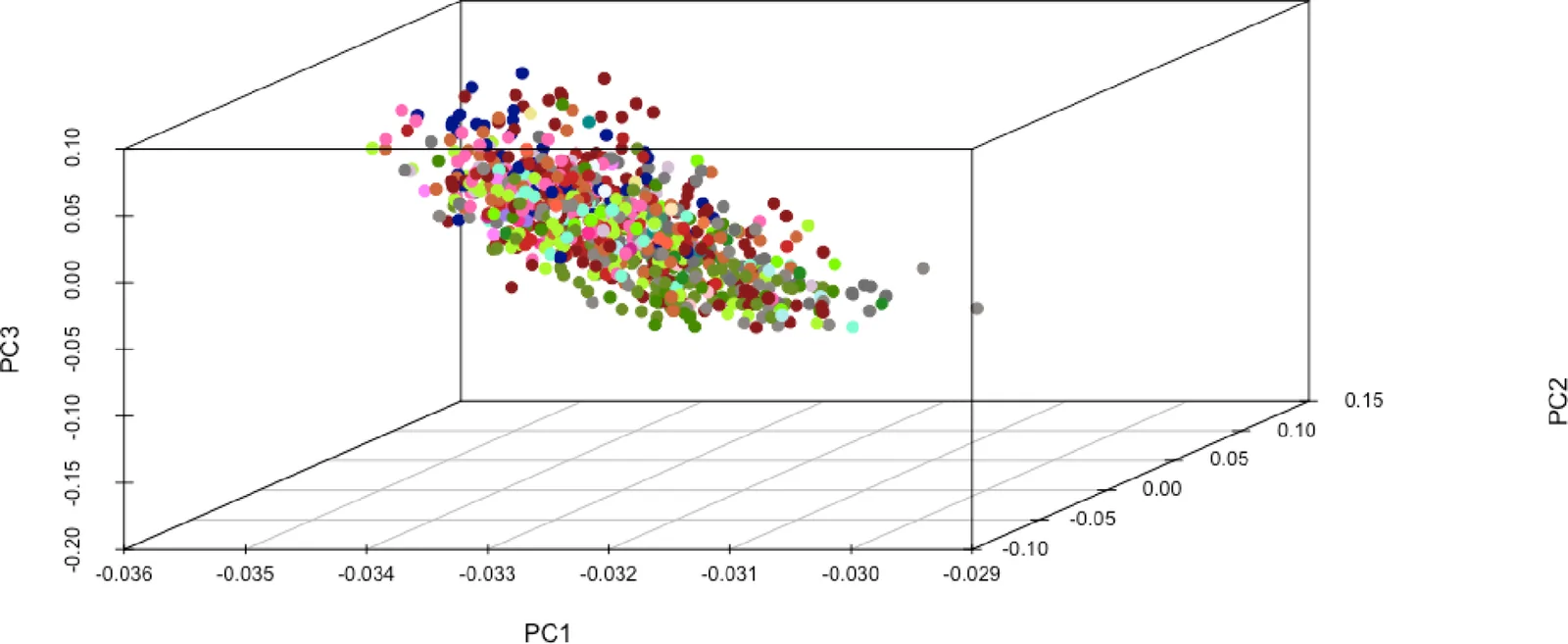
3-d plot of principal component analysis. Each dot represents a cow (n = 960) and each color represents a herd (n = 31)

**Fig. 2:**
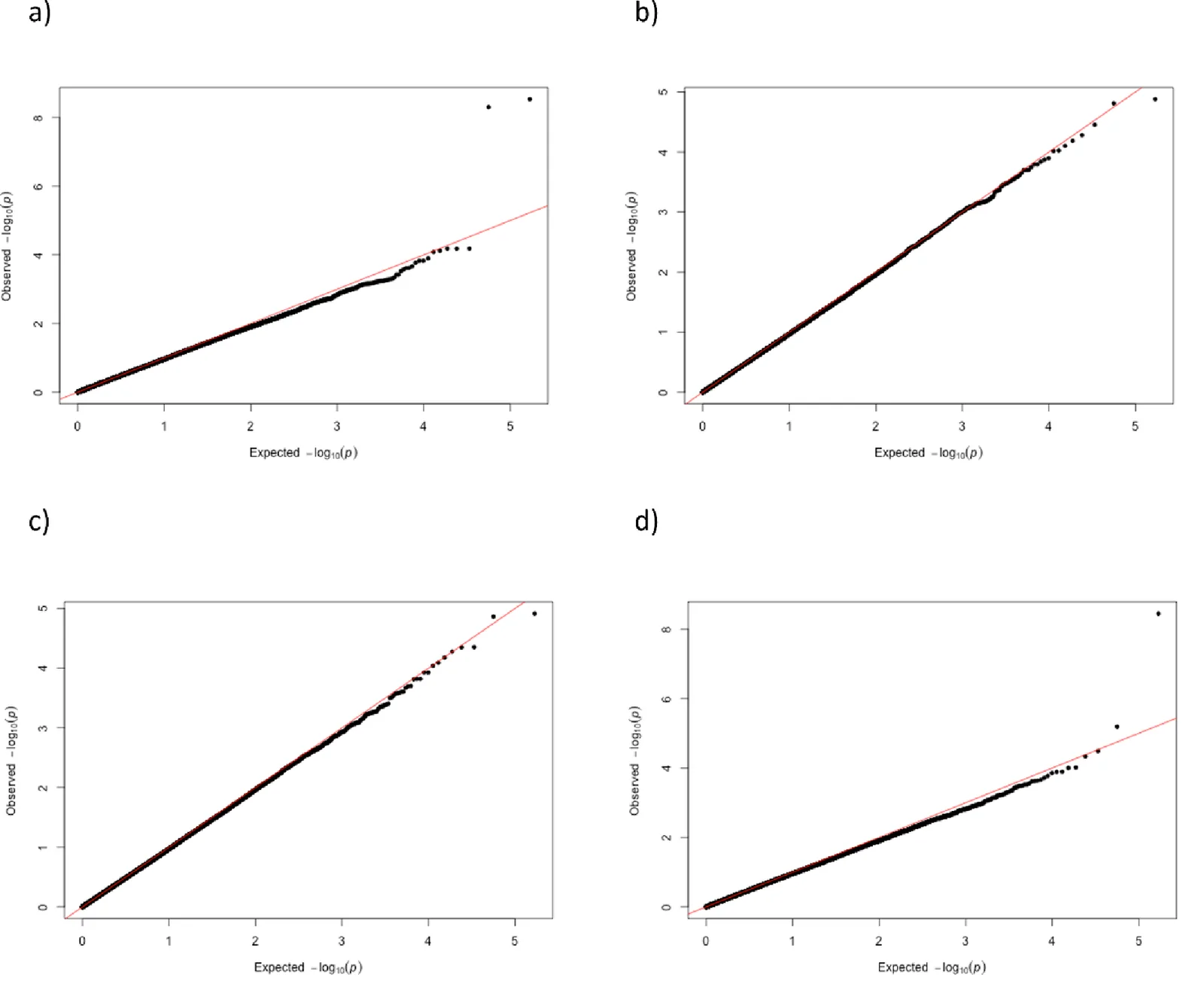
Quantile–quantile plot for the colostrum traits investigated. The panels **a**, **b**, **c**, and **d** refer to colostrum yield (L), immunoglobulin G concentration (g/L), total immunoglobulin concentration (g/L), and immunoglobulin G yield (g), respectively

The SNP genotypes were used for the principal component analysis (Fig. 1) to evaluate the presence of population structure. No subpopulations or genetic differences among farms were detected. Moreover, the quantile–quantile plots (Fig. 2) revealed no systematic bias and indicated that the model in Eq. (1) adequately fitted the data.

**Fig. 3:**
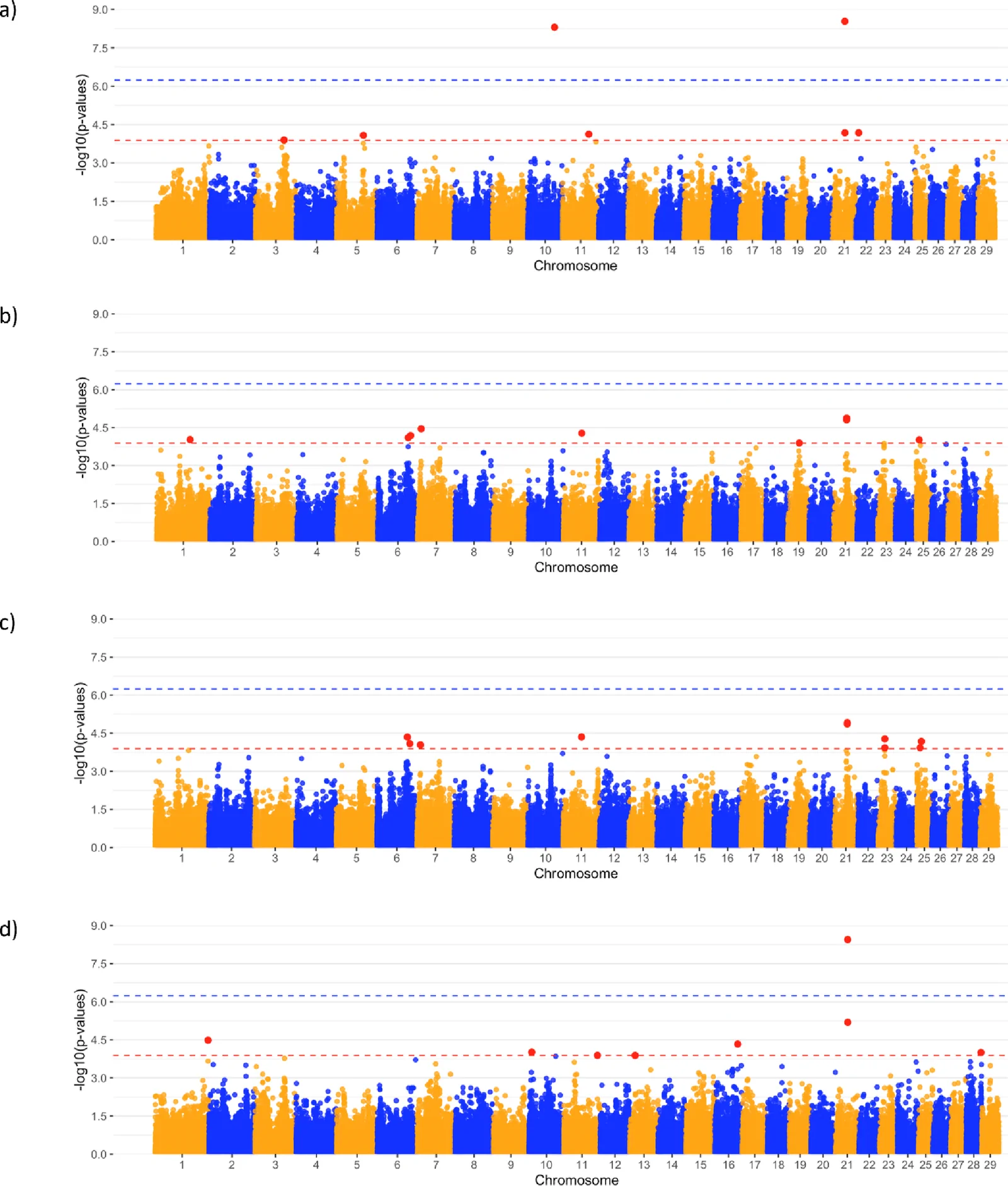
Manhattan plot for the colostrum traits investigated. The panels **a**, **b**, **c**, and **d** refer to colostrum yield (L), immunoglobulin G concentration (g/L), total immunoglobulin concentration (g/L), and immunoglobulin G yield (g), respectively. Significant SNPs above the FDR threshold (blue dashed line) are reported in red. Interactive.*html* versions of the Manhattan plots are available as supplementary material [Additional files 4–7, Figures S6–S9]

Relevant QTL containing the significant markers for the four traits investigated are graphically reported in the Manhattan plots in Fig. 3 and are listed in Tables 2, 3, 4 and 5. Only few significant SNPs were intragenic as most of them were nearby multiple potential candidate single genes or families.

**Table 2:** Significant SNP name, position, significance (*P*), and gene(s) identified for colostrum yield

SNP	BTA	bp	*P*	Gene symbol (± 0.5 Mb)
BTA-68578-no-rs	3	84,524,709	1.27E-04	*LOC100847503, LOC101906939, LOC104971723, LOC104971724, LOC112445885, LOC112445910, NFIA*^*a*^*, TRNAG-CCC*
BovineHD0500022567	5	79,144,129	8.28E-05	*CAPRIN2, IPO8, LOC104972518, LOC132345389, LOC132345401, LOC132345402, SINHCAF*
Hapmap26419-BTA-147498	10	79,089,147	4.94E-09	*ARG2, ATP6V1D, EIF2S1, GARIN2, GPHN*^*a*^*, LOC101905550, LOC104973218, LOC132346402, LOC132346403, PALS1, PIGH, PLEK2, PLEKHH1, RDH11, RDH12, TMEM229B, VTI1B*
ARS-BFGL-NGS-113740	11	77,601,906	7.50E-05	*APOB, LOC101902225, LOC112448818*^*a*^*, LOC513245, LOC785416, MIR2285AF-1, TDRD15, TRNAC-ACA, TRNAC-GCA*
BTB-01525804	21	36,267,216	2.92E-09	*LOC112443154, LOC132343365*^*a*^*, LOC788748, NOVA1*
BTB-01526059	21	36,498,605	6.63E-05	*LOC132343365, LOC788748, NOVA1*
ARS-BFGL-NGS-114164	22	7,687,485	6.61E-05	*CCR4, CLASP2, CRTAP, FBXL2, GLB1, LOC100847917, LOC107131651, LOC112443433, LOC112443519, LOC112443562, LOC112443565, LOC132343477, LOC132343478, LOC132343479*^*a*^*, LOC785477, LOC789829, PDCD6IP, SUSD5, TMPPE, TRIM71, U6, UBP1*
BovineHD2200002331	22	7,721,915	6.61E-05	*CCR4, CLASP2, CRTAP, FBXL2, GLB1, LOC100847917, LOC107131651, LOC112443433, LOC112443519, LOC112443543, LOC112443562, LOC112443565, LOC132343477, LOC132343478, LOC132343479*^*a*^*, LOC785477, LOC789829, PDCD6IP, SUSD5, TMPPE, TRIM71, U6, UBP1*

^a^Intragenic, based on the *Bos Taurus* genome assembly ARS-UCD 1.3

**Table 3:** Significant SNP name, position, significance (*P*), and gene(s) identified for immunoglobulin G concentration

SNP	BTA	bp	*P*	Gene symbol (± 0.5 Mb)
BovineHD0100028511	1	99,142,635	9.45E−05	*EGFEM1*^*a*^*, GOLIM4, LOC104970976, LOC107132202, LOC132345742, LRRC77, MIR551B*
BTB-01657893	6	89,736,709	7.89E−05	*AREG, BTC, EPGN, EREG, LOC101907265, LOC112447165, LOC132345525, LOC783750, MTHFD2L, PARM1*
BovineHD0600027418	6	96,823,720	6.51E−05	*ENOPH1, HNRNPD, HNRNPDL, LOC100141086, LOC112447111, RASGEF1B*^*a*^*, TMEM150C, U4, VAMP9*
BovineHD0700003008	7	10,494,698	3.51E−05	*LOC100138633, LOC100299045, LOC100300051, LOC100336567, LOC100337213, LOC132345686, LOC132345687, LOC132345812, LOC780971, LOC788031, OR7A106, OR7A112, OR7A113, OR7A115, OR7A116, OR7A119, OR7A122, OR7A133P, OR7A143P, OR7A146P, OR7A148P, OR7A154P, OR7A157P, OR7A158P, OR7A163P, OR7A164P, OR7A166P, OR7A168P, OR7A171P, OR7A172P, OR7A173P, OR7A180P, OR7A282P, OR7A80, OR7A81P, OR7A82, OR7A84, OR7A86, OR7A88, OR7A91, OR7A94, OR7A96*^*a*^*, OR7W1P*
ARS-BFGL-NGS-7469	11	54,492,825	5.24E−05	*CTNNA2, LOC101903989, LOC132346498, U1*
BovineHD1900018894	19	33,879,564	1.27E−04	*ADORA2B, AKAP10, ALDH3A1, ALDH3A2, B9D1, EPN2, FAM83G, GRAP, LOC112442748, LOC132342975, LOC132343052, MAPK7, MFAP4, MIEF2, NCOR1, PRPSAP2, RNF112, SHMT1, SLC47A1, SLC47A2, SLC5A10, SMCR8, SPECC1, TOP3A, TRNAG-CCC, TRNAW-CCA, TTC19, ULK2, ZSWIM7*
BTB-01781411	21	38,734,297	1.54E−05	*LOC781164*
BTB-00818514	21	38,862,318	1.32E−05	*FOXG1, LOC101905308, LOC132343366, LOC781164*
BovineHD2500002292	25	8,646,027	9.63E−05	*GRIN2A*^*a*^*, LOC112444299, LOC132343840, TRNAG-CCC, U6*

^a^Intragenic, based on the *Bos Taurus* genome assembly ARS-UCD 1.3

**Table 4:** Significant SNP name, position, significance (*P*), and genes identified for total immunoglobulin concentration

SNP	BTA	bp	*P*	Gene symbol (± 0.5 Mb)
BTB-01657893	6	89,736,709	4.48E−05	*MTHFD2L, EPGN, EREG, LOC112447165, LOC132345525, AREG, BTC, LOC101907265, PARM1, LOC783750*
BovineHD0600027418	6	96,823,720	8.12E−05	*RASGEF1B*^*a*^*, LOC112447111, VAMP9, HNRNPD, LOC100141086, HNRNPDL, ENOPH1, TMEM150C, U4*
BovineHD0700003008	7	10,494,698	9.09E−05	*LOC788031, LOC132345686, OR7A172P, OR7A88, OR7A154P, OR7A163P, OR7A112, OR7A106, OR7A282P, OR7A173P, OR7A122, OR7A168P, OR7A82, OR7A80, OR7A116, OR7A119, LOC100138633, OR7A81P, OR7A146P, OR7W1P, OR7A84, OR7A94, OR7A158P, OR7A171P, OR7A86, OR7A164P, OR7A166P, OR7A115, OR7A113, OR7A133P, LOC132345687, OR7A143P, OR7A91, OR7A96*^*a*^*, OR7A148P, LOC100337213, OR7A157P, OR7A180P, LOC780971, LOC100299045, LOC100300051, LOC100336567, LOC132345812*
ARS-BFGL-NGS-7469	11	54,492,825	4.44E−05	*LOC101903989, U1, CTNNA2, LOC132346498*
BTB-00818514	21	38,862,318	1.22E−05	*LOC781164, FOXG1, LOC132343366, LOC101905308*
BovineHD2300004847	23	19,140,564	1.19E−04	*SUPT3H, RUNX2, TRNAE-UUC, TRNAG-UCC, LOC112443836, LOC132343580, CLIC5, U6, ENPP4, ENPP5, RCAN2*
BovineHD2300004852	23	19,161,517	5.29E−05	*RUNX2, TRNAE-UUC, TRNAG-UCC, LOC112443836, LOC132343580, CLIC5*^*a*^*, U6, ENPP4, ENPP5, RCAN2*
BovineHD2500002292	25	8,646,027	1.18E−04	*TRNAG-CCC, GRIN2A*^*a*^*, LOC112444299, U6, LOC132343840*
Hapmap57812-rs29010040	25	12,352,826	6.64E−05	*SHISA9, LOC132343905, LOC132343843, ERCC4*

^a^Intragenic, based on the *Bos Taurus* genome assembly ARS-UCD 1.3

**Table 5:** Significant SNP name, position, significance (*P*), and gene(s) identified for immunoglobulin G yield

SNP	BTA	bp	*P*	Gene symbol (± 0.5 Mb)
ARS-BFGL-NGS-27660	1	156,207,768	3.26E−05	*KCNH8, MIR2285E-1, bta-mir-2285e-1, LOC112447008*
ARS-BFGL-NGS-115023	10	8,547,033	9.62E−05	*S100Z, LOC101907313, CRHBP, AGGF1, ZBED3, LOC107132813, LOC112448625, SNORA47, PDE8B*^*a*^*, LOC112448612, WDR41, OTP, TBCA, ATXN3*
BovineHD1100028768	11	98,945,411	1.29E−04	*SH2D3C, CDK9, FPGS, ENG, AK1, ST6GALNAC6, ST6GALNAC4, PIP5KL1, DPM2, LOC132346636, EEIG1, NAIF1, SLC25A25, PTGES2, LCN2, BBLN, CIZ1, LOC104973485, DNM1, MIR199B, bta-mir-199b, MIR3604-2, MIR3154, bta-mir-3154, GOLGA2, LOC132346638, SWI5, TRUB2, COQ4, TRNAR-UCU, SLC27A4, LOC132346637, URM1, IR219-1, bta-mir-219–1, MIR219B, U3, TRNAW-CCA, CERCAM, LOC112448915, SNORA70, ODF2, GLE1, SPTAN1, DYNC2I2, SET, PKN3, ZDHHC12, LOC101908339, ZER1, TBC1D13, U6, LOC104973489, ENDOG, SPOUT1, LOC132346505, KYAT1, LOC104968656, LRRC8A, PHYHD1, DOLK*
ARS-BFGL-BAC-13110	13	15,846,085	1.29E−04	*LOC104973714, GATA3, LOC112449253, TAF3, LOC112449254, ATP5F1C, KIN, ITIH2, LOC100138864, ITIH5, LOC107133028, LOC101903501, LOC112449255*
BTB-00656184	16	67,244,107	4.64E−05	*HMCN1*^*a*^*, LOC100138126, PRG4, TPR, LOC107133263, ODR4, PDC, TRNAW-CCA, PTGS2*
BTB-01525804	21	36,267,216	3.57E−09	*LOC112443154, LOC132343365*^*a*^*, LOC788748, NOVA1*
BovineHD2800012867	28	44,190,953	9.94E−05	*ERCC6, U6, SLC18A3, CHAT, C28H10orf53, LOC132344154, OGDHL, PARG, TIMM23, SNORA74, NCOA4, LOC132344155, MSMB, LOC100848205, WASHC2A, ZFAND4*^*a*^*, MARCHF8, LOC112444772, ALOX5, OR13A1, OR6D16, OR6D20P, OR6D18, OR6D19, OR6D26P, OR6D17, OR6D27P, ZNF22, RASSF4, DEPP1, TMEM72*

^a^Intragenic, based on the *Bos Taurus* genome assembly ARS-UCD 1.3

For CY, the most significant SNPs were located within the gene *GPHN* on BTA10 and the gene *NOVA1* on BTA21. Several signals, however, fell in LOC segments, usually regions still with an unclear role. Overall, the polygenic nature of CY is confirmed by presence of signals in BTA3, 5, 10, 11, 21, and 22. In particular, an important region was detected on BTA22, with the marker BovineHD2200002331 being within the annotated *LOC132343479* and flanking 22 genes. Similarly, the IgG concentration was associated with signals in BTA1, 6, 7, 11, 19, 21, and 25, with BovineHD0700003008 on BTA7 having the greatest number of accompanying genes. The degree of overlap differed markedly among traits, reflecting their distinct nature and polygenic architecture.

As expected from their strong phenotypic correlation (Table 1), IgG and IgTOT showed the highest overlap in significant signals: most IgTOT-associated SNPs were also detected for IgG concentration, whereas a limited number of SNPs were trait-specific. In contrast, IgG yield showed limited overlap with both CY and IgG concentration, despite its mathematical dependence from these two traits and their correlations (Table 1). Only BTB-01525804 on BTA21 was shared between CY and IgG yield.

Two markers on BTA23 and a marker on BTA25 were detected for IgTOT only. The phenotype with the lowest number of significant markers was IgG yield, which shared only one out of the 7 SNPs. However, the number of intragenic signals did not differ, with 5, 4, 4, and 4 SNPs falling within a coding region for CY, IgG, IgTOT, and IgG yield, respectively.

**Fig. 4:**
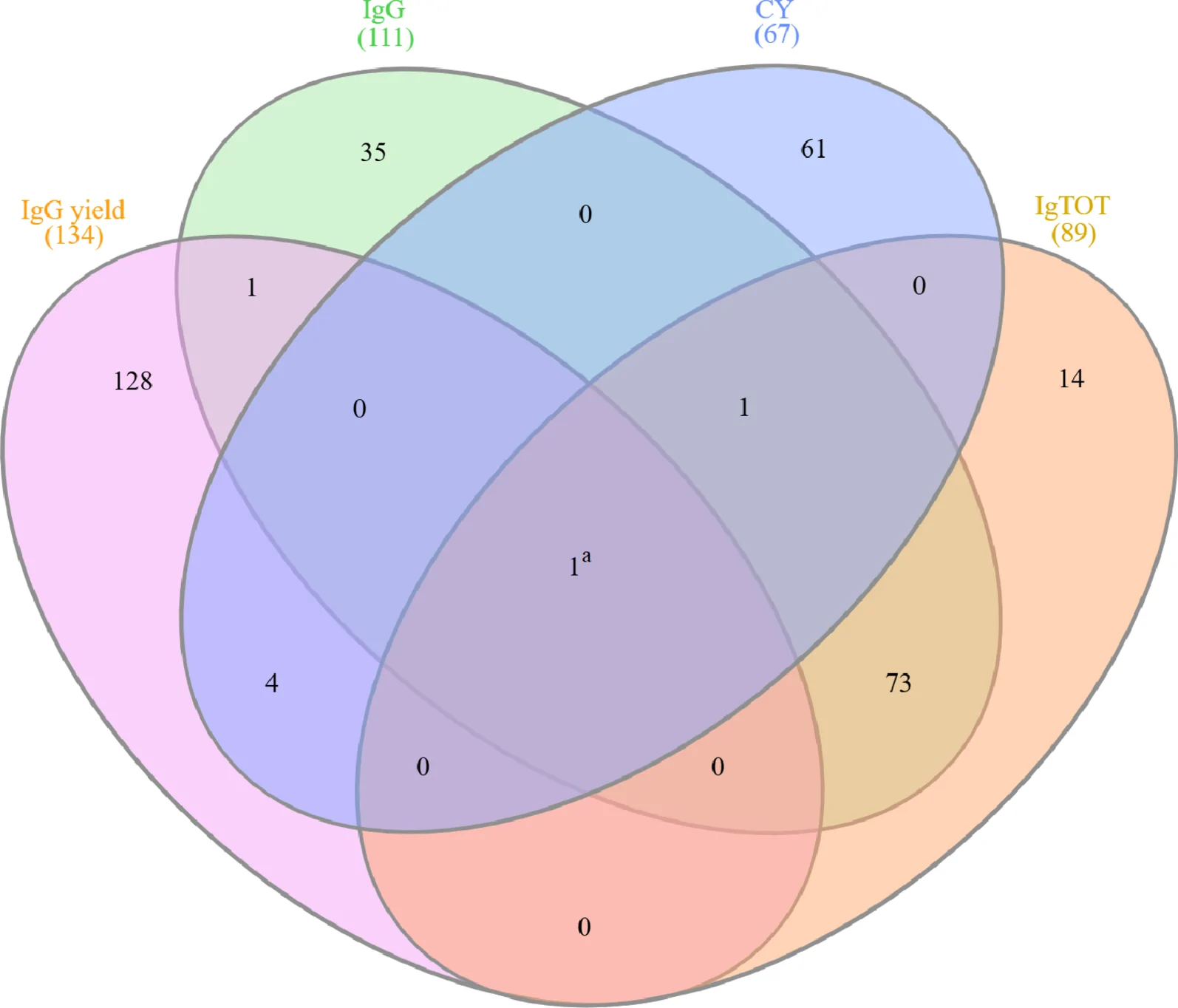
Venn diagram for the significant genes (± 0.5 Mb) of the colostrum traits investigated. CY = colostrum yield (L); IgG = immunoglobulin G concentration (g/L); IgTOT = total immunoglobulin concentration (g/L); IgG yield = immunoglobulin G yield (g). ^a^ Shared *U6* annotation

The Venn diagram (Fig. 4) was intended to show overlaps of annotated genes within the selected genomic windows for the traits investigated, without necessarily indicating shared causal genes or evidence of a common functional mechanism. U6 annotations were identified among the genomic regions detected for the traits.

### Exploratory functional enrichment analysis

The GO terms and the KEGG pathways found in this preliminary study are listed in the supplementary table S2 [Additional file 3, Table S2] and their graphical overview are presented as bubble plots [Additional files 4–7, Figures S2-S5].

For CY, none of the 7 terms surpassed the significance threshold (FDR < 0.05) and, considering the number of genes, the major terms were GO:0005739 ~ mitochondrion with 11 genes enriched and KEGG bta01100:metabolic pathways with 15 genes enriched. A total of 23 GO terms and three KEGG pathways were associated with the IgG concentration, but only 11 of them had FDR < 0.05, of which only one was a KEGG. For this trait, the top three significant pathways in terms of number of genes were the GO:CC 0016020 ~ membrane, which represented 32 genes, the GO MF:0004930 ~ G protein-coupled receptor activity which represented 21 genes, and the GO BP:0007186 ~ G protein-coupled receptor signaling pathway which represented 18 genes. Although the number of total pathways identified for IgTOT was 18, only 11 were significant. In addition, a tendency was found for the GO:BP:0007166 (“cell surface receptor signaling pathway”; FDR = 0.07) and the GO:BP:0038134 (“ERBB2-EGFR signaling pathway”; FDR = 0.10). Moreover, 28 genes were associated with the term GO CC:0016020 ~ membrane and 20 associated with the term GO BP:0004930 ~ G protein-coupled receptor activity. Like CY, all the 22 IgG yield terms had FDR ≤ 0.05, but the KEGG pathway “bta01100:metabolic pathways” was the term associated with the greatest number of genes (n = 15).

## Discussion

The present study aimed at elucidating the genomic architecture of colostrability (sufficient CY and IgG) and exploring the possibility of evaluating animals according to their polygenic predisposition to yield a sufficient amount of good-quality colostrum at first milking. Phenotypes analyzed in this study were retrieved from a dataset previously used to estimate pedigree-based genetic parameters of colostrum traits in 2938 Italian Holstein cows [11], whose descriptive statistics, distributions, and correlations were consistent with previous studies on dairy cattle colostrum [24]. In that study, the phenotypic (-0.219 ± 0.032) correlation between CY and IgG concentration is in line with the Pearson correlation observed here (Table 1), further supporting the presence of a dilution effect.

The genomic regions identified in the present study should be considered novel mainly with respect to colostrability. Some of these regions may overlap with, or be located near, QTL previously reported for other quantitative traits in cattle. Therefore, the absence of previous GWAS on exactly the same phenotypes does not imply that all associated regions are completely unexplored in the bovine QTL literature. Despite the limited number of studies investigating the genetic basis of colostrum traits in dairy cattle, comparisons with the available literature remain highly valuable. Existing studies provide important reference points for interpreting newly identified genomic regions and for assessing the consistency of associations across populations and experimental settings. Such comparisons are particularly relevant for traits related to colostrum quality, circulating IgG concentration, and immunity, which are complex biological phenotypes that remain largely unexplored from a genomic perspective. Nevertheless, the interpretation of similarities and discrepancies among studies should take into account several methodological differences. Most previous investigations relied on the refractive index as a proxy for colostrum quality rather than on directly measured IgG concentration. Furthermore, for both colostrum yield and antibody concentration, sampling time and sampling protocols—which are major sources of phenotypic variation—often differed across studies. For example, colostrum yield may have been recorded at the first or subsequent milkings, samples collected either at calving or several days later, and IgG quantified using different analytical methods such as ELISA or radial immunodiffusion. Additional sources of variation include differences in population structure, genetic background, phenotypic variability, and reference genome assembly. Therefore, direct comparisons should be interpreted with caution. However, rather than diminishing their relevance, these differences highlight the need for further investigations aimed at validating and refining candidate genes, haplotypes, and genomic regions involved in colostrum traits and immune-related functions.

The Manhattan plots (Fig. 3) graphically confirmed the polygenic nature of the four colostrability traits, with numerous significant signals spread across the genome. The*.html* interactive versions of Manhattan plots are available as supplementary files [Additional files 8–11, Figures S6-S9]. For CY, the two most significant SNPs were found in *GPHN* and *NOVA1*, which are located on different autosomes. *GPHN* is responsible for the synthesis of molybdenum, which is fundamental for enzymes synthesis. Together with *NFIA*, which hosts the significant SNP BTA-68578-no-rs and is in charge of immune cells development and mammary cells proliferation, *GPHN* can be involved in the metabolic regulation of mammary gland functions. *NOVA1*, on the other hand, is reported to be a hormone regulator in mice and to act in the regulation of metabolic process. This gene was previously reported by Raschia et al. 2020 [25], who carried out a GWAS for different cow milk traits.

The most significant signal of the IgG concentration flanked the *FOXG1*, a transcription factor on BTA21 that encodes for proteins fundamental for brain cells growth and is responsible for brain anomalies [26]. This gene, in fact, is widely known for mutations that cause neurological disorders in various species, including the FOXG1-syndrome in humans [27].

Several signals were within or near genes belonging to the olfactory receptor 7 subfamily A (*OR7A*), a complex located in the BTA7. In fact, both IgG and IgTOT concentrations had the same intragenic significant SNP in the *OR7A96* region, whose genic product is expressed in the olfactory epithelium and is responsible for the detection of specific odorant molecules. There is no evidence regarding the exact types of molecules that these receptors detect and *OR7A96* has never been identified in previous GWAS on cattle. Given the exploratory nature of this study and the results of functional enrichment analysis, the presence of a positional signal may only be due to proximity to a clustered olfactory receptor gene family. It is instead plausible that this olfactory receptor gene-family affects other important phenotypes in dairy cattle with a direct biological function, such as reproductive behavior (hormones), communication (pheromones), and feeding selection and intake (feed volatile compounds).

Similarly, *GRIN2A* and *RASGEFF1B* contained a significant signal for both IgG and IgTOT. The first is a glutamate ionotropic receptor known in humans for its mutations, which cause neurodevelopmental and neurological issues, including epilepsy and speech disorders. Similarly, recent studies in humans suggest that *RASGEF1B* plays a role in the central nervous system too, affecting neurochemistry and affecting behavior and cognition. Overall, this gene regulates cellular signaling pathways: its genic product, in fact, a guanine nucleotide exchange factor, activates Ras-like GTPases, which act as molecular switches. Notably, it has been reported to be involved in the regulation of gene expression in macrophages, with upregulation in response to bacterial stimuli such as the presence of lipopolysaccharides, an endotoxin found in the outer membrane of gram-negative bacteria. In particular, it influences both steady-state (baseline) and signal-dependent expression of genes, thereby affecting inflammatory and immune responses in macrophages. In the same region, *LRRC77* and *MIR551B* were detected. While the first was associated with behaviour and mental health in a murine model, the second was significant in GWAS for calving ease in Nellore cattle [28]. Other relevant genes have been reported to be significant in cattle, e.g., *GOLIM4* for feed efficiency [29], *EREG* and *AREG* for follicular development [30], coat color [31], and morphology [32], *AREG* for immunological disease and inflammatory response [33], and *EREG*, *AREG*, and *BTC* for twinning [34]. *ENOPH1* has been recently identified in a study on dairy cow resistance to mastitis in which the authors concluded that it may play a role in the immune response in bovines [35]. In addition, this gene was reported as associated with stress response in mice [36].

Moreover, one of the genes identified in BTA6 (*MTHFD*2L) was significant in a Canadian study on the milk concentration of beta-hydroxybutyrate in Holstein cows [37] and in a multibreed Irish study on cattle carcass traits [38]. The literature indicates that *PARM1* is involved in the development of blastocysts [39], whereas *CTNNA2* has been reported as a key-gene for calf mortality in Nellore breed [40]. The same gene is also near regions associated with female longevity in beef cattle [41] and fell into a QTL for myopathies in the Piedmontese breed [42].

In this study, the signal in BTA19 for IgG was very close to genes previously reported for milk fatty acid composition of Belgian Blue cows [43], including *MIEF2*, which has been reported as significant coding region for carcass traits in Nellore cattle [44].

Very few GWAS for IgG in colostrum or serum have been carried out in dairy and beef cattle. As previously indicated, in some cases, the absence of overlapping regions can be attributed to the different methods used to measure CY and determine colostrum quality. The latter, in fact, has been often assessed indirectly, through the refractive index rather than the real antibodies concentration.

The refractive index, expressed in BRIX degrees, represents the total solids concentration of a semisolid matrix, therefore, it is not perfectly correlated with the real colostral IgG concentration. The two traits are different at both phenotypic and genetic levels, making direct comparison between them rather unfair. In addition, previous studies have widely demonstrated that sampling time strongly affects phenotypic and genetic variability of colostrum traits. In fact, IgG concentration and CY change dramatically in the first hours after calving, with decreasing antibodies concentration and increasing udder secretion. In Greek Holstein, Soufleri et al. (2019) [5] investigated the refractive index in colostrum samples collected up to 16 h post-partum, whereas other studies are based on protocols in which colostrum had to be collected within 6 h postpartum at first milking and IgG was directly determined with a reference method [8].

In Chinese Holsteins, the genomic background of IgG concentration and different immunoglobulin isotypes in calf serum and colostrum determined via ELISA has been investigated [45]. Significant or suggestive signals were reported on BTA3, BTA6, and BTA22 for serum IgG-related traits and on BTA2, BTA4, BTA6, BTA18, and BTA22 for colostrum IgG-related traits [45]. Some of these chromosomes, particularly BTA6 and BTA22, were also detected in the present study, although no clear exact positional or candidate-gene overlap was observed for IgG concentration or IgTOT. For colostrum IgG, the main candidate genes reported in Chinese Holsteins included *NCF1*, *IKBKG*, *FGFR4*, *FGFR2*, *SORBS3*, and *IGHV1S18* [45]. Interestingly, *PTGS2*, another gene reported by Lin et al. [45] for immunoglobulin-related traits, was located within the genomic window associated with IgG yield in the present study; however, this overlap involved a derived trait and should be interpreted cautiously. In the same population, a genomic investigation of colostrum refractive index identified parity-specific signals on BTA6, BTA9, BTA19, and BTA22 [46]. Similarly, a GWAS for immunoglobulin isotypes in Swedish dairy cattle detected regions for milk IgG1 on BTA3 and BTA21, for total IgG in colostrum on BTA9, BTA11, and BTA20, and for colostrum refractive index on BTA3 [47]. Overall, an apparent chromosome-level convergence for immunoglobulin-related traits exists in dairy cattle, particularly on BTA6, BTA11, BTA21, and BTA22. However, the lack of clear direct replication at the positional or candidate-gene level indicates that comparisons should be interpreted cautiously, likely due to differences in population, phenotype assessment and definition, sampling protocol, and statistical approach.

The candidate genes of CY were enriched with intracellular protein transport and trafficking, which are crucial for protein secretion and can influence mammary gland secretion yield . In addition, the most significant pathway was related to retinoid metabolism which plays a role in epithelial differentiation, reasonably including in the mammary gland epithelium. On the other hand, IgG concentration was enriched with immune-related processes, signaling, and epithelial receptor pathways . In fact, many olfactory receptors, which are expressed in immune cells and epithelial cells, are involved in signaling and pathogens recognition and the epidermal growth factor receptor (i.e., *EGFR*) has been reported to be critical for immune modulation in secretory tissues.

Overall, the analysis revealed that CY and IgG concentration have different biological layers, one more structural and one more functional, respectively. The transfer of antibodies to colostrum seems to be strongly influenced by receptor-mediated processes. Figure 4 shows that all traits share a common annotation, *U6*, which is known as a noncoding segment generally repeated across the genome in many species and is essential for the RNA splicing after transcription. Therefore, *U6* should not be considered as a proper candidate coding region as there is no evidence of a shared functional mechanism across the colostrability traits; causality could not be verified in this study. Nevertheless, the involvement of this region in RNA processing is relevant for the regulation of biological processes. In both cases, some of the pathways converged to trafficking and signaling. However, these results should be interpreted carefully in the light of the fact that refractive index instead of IgG concentration was used and, last, that Italian and Chinese Holstein populations are very likely to be significantly distant from each other.

Finally, none of the significant SNPs and no signals’ peaks were detected in major well-known milk-production genes [48], e.g., *DGAT1* (BTA14), *GHR* (BTA20), *PRLR* (BTA20), *ABCG2* (BTA6), the casein cluster (BTA6), *SCD1* (BTA26), *FASN* (BTA19), and *AGPAT6* (BTA27).

Overall, results support the idea that regions affecting both yield and composition of mature milk differ from those governing colostrogenesis and IgG concentration in the immediate post-partum. In fact, genes associated with milk traits are reported to be in charge of regulation of fat synthesis and protein content, and prolactin release and persistence, which are essential for lactogenesis. Therefore, colostrability appears to be regulated by distinct biological domains, largely independent from the genetic architecture of mature milk. On the other hand, although the dataset is valuable given the difficulty of phenotyping the colostrability at scale, the sample size remains limited, making this study a basis for future deeper investigations on colostrability biological causal mechanisms.

## Conclusions

In the present study, the genetic determinism of colostrability was explored through GWAS in Italian Holsteins. The results confirmed the complex genetic background of these phenotypes, with significant signals scattered across the genome, including some regions potentially involved in immune response regulation. The absence of major known QTLs with large and consistent effects does not reduce the relevance of these findings, but rather supports the highly polygenic nature of the traits and the need for larger, harmonized datasets for validation, to better resolve their genomic architecture. The functional enrichment analysis, in fact, provided preliminary biological context for the detected regions, making this study a basis for future investigations at the population level. Although colostrum-related phenotypes have been collected using different protocols across studies, aggregation of CY and IgG concentration or refractive index data from multiple phenotyped populations is advisable to improve dataset size. From this perspective, adopting a standardized sampling protocol in future studies would be essential to ensure data comparability. Colostrability is a promising set of traits for the future development of a specific calf health index in addition to neonatal traits such as the absorption capacity of the gut epithelium, permeability to colostrum bioactive factors and nutrients, resistance to disease, and survival in the first days of life.

## Supplementary Information

Below is the link to the electronic supplementary material.

**Additional file 1: Fig. S1.** Data distribution for the colostrum traits investigated**. **The panels a), b), c), and d) refer to colostrum yield (L), immunoglobulin G concentration (g/L), total immunoglobulin concentration (g/L), and immunoglobulin G yield (g), respectively.

**Additional file 2: Table S1.** Average of the colostrum traits investigated across the herds.

**Additional file 3: Table S2.** Functional enrichment analysis terms for the colostrum traits investigated.

**Additional file 4: Fig. S2.** Functional enrichment analysis bubble plot for colostrum yield.

**Additional file 5: Fig. S3.** Functional enrichment analysis bubble plot for immunoglobulin G concentration.

**Additional file 6: Fig. S4.** Functional enrichment analysis bubble plot for total immunoglobulin concentration. Terms source: gene ontology cellular components, biological processes, and molecular function and Kyoto Encyclopedia of Genes and Genomes pathways. For all terms: *P *≤ 0.10 and no restriction was done based on the FDR.

**Additional file 7: Fig. S5.** Functional enrichment analysis bubble plot for immunoglobulin G yield. Terms source: gene ontology cellular components, biological processes, and molecular function and Kyoto Encyclopedia of Genes and Genomes pathways. For all terms the *P*-value was equal or below 0.10 and no restriction was done based on the FDR.

**Additional file 8: Fig. S6.** Interactive Manhattan plot for colostrum yield.

**Additional file 9: Fig. S7.** Interactive Manhattan plot for immunoglobulin G concentration.

**Additional file 10: Fig. S8.** Interactive Manhattan plot for total immunoglobulin concentration.

**Additional file 11: Fig. S9.** Interactive Manhattan plot for immunoglobulin G yield.

## Data Availability

The datasets generated and/or analysed during the current study are available from the corresponding author upon request.
