# Supplementary material for: Genome-wide association study reveals key genomic regions underlying colostrability in Italian Holstein cattle
Source: Genet Sel Evol. 2026 Aug 20;58:54. doi: 10.1186/s12711-026-01080-7 (PMC13491712; doi:10.1186/s12711-026-01080-7)
Supplement: Supplementary file 1 [file 12711_2026_1080_MOESM2_ESM.pdf]

Table S1 Average of the colostrum traits investigated across the herds

Herd	CY	IgG	IgTOT	IgG yield
A1	3.00	144.61	163.25	304.08
A2	4.86	97.32	105.45	504.74
A3	3.83	93.40	106.68	351.29
A4	5.60	130.60	141.25	652.75
A5	3.00	119.34	135.97	358.02
A6	3.13	120.72	135.61	376.88
A7	3.75	104.01	119.83	435.68
A8	4.14	100.05	115.52	412.62
A9	5.00	42.00	51.38	210.00
A10	5.50	93.23	102.28	504.75
A11	13.67	97.43	101.78	1303.85
A12	4.68	109.42	124.26	467.62
A13	4.00	77.91	88.21	320.77
A14	7.00	124.46	135.68	871.22
A15	9.60	71.19	86.18	577.57
A16	2.67	142.05	163.36	363.87
A17	6.25	73.17	93.51	466.61
A18	4.18	102.71	115.00	404.06
A19	7.33	101.17	115.22	733.74
A20	3.83	103.08	117.30	375.74
A21	3.17	78.74	91.39	223.61
A22	5.30	97.67	107.69	520.40
A23	4.55	118.66	131.37	511.65
A24	2.50	139.67	158.26	349.17
A25	5.74	100.19	111.26	571.65
A26	4.79	117.79	131.61	538.99
A27	4.66	115.51	129.40	507.08
A28	5.21	94.96	108.82	487.08
A29	4.00	102.44	114.84	396.29
A30	3.80	104.19	118.37	383.67
A31	6.08	80.66	95.85	484.17

CY = colostrum yield (L); IgG = immunoglobulin G concentration (g/L); IgTOT = total immunoglobulin concentration (g/L); IgG yield = immunoglobulin G yield (g)
Raw means provided only as descriptive information; herd-season was included in the GWAS model to account for management and environmental differences.
N of observations per herd ranged from 1 to 147.
