# Supplementary material for: Genome-wide association study reveals key genomic regions underlying colostrability in Italian Holstein cattle
Source: Genet Sel Evol. 2026 Aug 20;58:54. doi: 10.1186/s12711-026-01080-7 (PMC13491712; doi:10.1186/s12711-026-01080-7)
Supplement: Supplementary file 2 [file 12711_2026_1080_MOESM3_ESM.pdf]

Table S2 Functional enrichment analysis terms for the colostrum traits investigated

Trait	Category	Term[a]	N genes
CY	GO:MF	GO:0052650~all-trans-retinol dehydrogenase (NADP+) activity	2
	KEGG	bta01240:Biosynthesis of cofactors	3
	KEGG	bta01100:Metabolic pathways	7
	GO:BP	GO:0007283~spermatogenesis	3
	GO:BP	GO:0006886~intracellular protein transport	3
	GO:CC	GO:0005829~cytosol	8
	GO:CC	GO:0005794~Golgi apparatus	4
IgG	GO:MF	GO:0004930~G protein-coupled receptor activity	21
	GO:MF	GO:0004984~olfactory receptor activity	17
	GO:MF	GO:0005154~epidermal growth factor receptor binding	5
	GO:BP	GO:0007186~G protein-coupled receptor signaling pathway	18
	GO:BP	GO:0045741~positive regulation of epidermal growth factor-activated receptor activity	4
	KEGG	bta04740:Olfactory transduction	17
	GO:BP	GO:0045840~positive regulation of mitotic nuclear division	4
	GO:CC	GO:0016020~membrane	32
	GO:BP	GO:0007173~epidermal growth factor receptor signaling pathway	4
	GO:BP	GO:0007165~signal transduction	8
	GO:BP	GO:0007166~cell surface receptor signaling pathway	5
	GO:MF	GO:0140968~polyspecific organic cation:proton antiporter activity	2
	GO:MF	GO:0008083~growth factor activity	4
	GO:MF	GO:0004028~3-chloroallyl aldehyde dehydrogenase activity	2
	GO:BP	GO:0038134~ERBB2-EGFR signaling pathway	2
	GO:BP	GO:1990961~xenobiotic detoxification by transmembrane export across the plasma membrane	2
	GO:BP	GO:0006081~aldehyde metabolic process	2
	GO:MF	GO:0030297~transmembrane receptor protein tyrosine kinase activator activity	2
	GO:BP	GO:0035999~tetrahydrofolate interconversion	2
	GO:MF	GO:0004029~aldehyde dehydrogenase (NAD+) activity	2

	KEGG	bta04012:ErbB signaling pathway	3
	GO:MF	GO:0042910~xenobiotic transmembrane transporter activity	2
	GO:MF	GO:0016620~oxidoreductase activity, acting on the aldehyde or oxo group of donors, NAD or NADP as acceptor and acting as single donors with incorporation of molecular oxygen	2
	GO:BP	GO:0008284~positive regulation of cell population proliferation	4
	GO:CC	GO:0005886~plasma membrane	22
	KEGG	bta00340:Histidine metabolism	2
IgTOT	GO:MF	GO:0004930~G protein-coupled receptor activity	20
	GO:MF	GO:0004984~olfactory receptor activity	17
	GO:BP	GO:0007186~G protein-coupled receptor signaling pathway	18
	KEGG	bta04740:Olfactory transduction	17
	GO:BP	GO:0045741~positive regulation of epidermal growth factor-activated receptor activity	4
	GO:CC	GO:0016020~membrane	28
	GO:BP	GO:0045840~positive regulation of mitotic nuclear division	4
	GO:MF	GO:0005154~epidermal growth factor receptor binding	4
	GO:BP	GO:0007173~epidermal growth factor receptor signaling pathway	4
	GO:BP	GO:0007165~signal transduction	7
	GO:MF	GO:0008083~growth factor activity	4
	GO:BP	GO:0007166~cell surface receptor signaling pathway	4
	GO:BP	GO:0038134~ERBB2-EGFR signaling pathway	2
	GO:MF	GO:0030297~transmembrane receptor protein tyrosine kinase activator activity	2
	GO:BP	GO:0008284~positive regulation of cell population proliferation	4
	KEGG	bta04012:ErbB signaling pathway	3
	GO:CC	GO:0005886~plasma membrane	17
	GO:MF	GO:1990837~sequence-specific double-stranded DNA binding	2
IgG yield	GO:CC	GO:0005739~mitochondrion	11
	GO:MF	GO:0016829~lyase activity	3
	KEGG	bta01100:Metabolic pathways	15
	GO:MF	GO:0016212~kynurenine-oxoglutarate transaminase activity	2
	GO:MF	GO:0001665~alpha-N-acetylgalactosaminide alpha-2,6-sialyltransferase activity	2

GO:BP	GO:0001574~ganglioside biosynthetic process	2
GO:BP	GO:0030212~hyaluronan metabolic process	2
GO:MF	GO:0016702~oxidoreductase activity, acting on single donors with incorporation of molecular oxygen, incorporation of two atoms of oxygen	2
GO:BP	GO:0001516~prostaglandin biosynthetic process	2
GO:CC	GO:0005743~mitochondrial inner membrane	5
GO:BP	GO:0009311~oligosaccharide metabolic process	2
KEGG	bta00590:Arachidonic acid metabolism	3
GO:MF	GO:0008373~sialyltransferase activity	2
GO:CC	GO:0008023~transcription elongation factor complex	2
GO:BP	GO:0006486~protein glycosylation	3
KEGG	bta00604:Glycosphingolipid biosynthesis - ganglio series	2
GO:MF	GO:0042803~protein homodimerization activity	5
GO:BP	GO:0046034~ATP metabolic process	2
KEGG	bta00450:Selenocompound metabolism	2
GO:CC	GO:0042995~cell projection	3
GO:BP	GO:0060261~positive regulation of transcription initiation by RNA polymerase II	2
GO:BP	GO:0009058~biosynthetic process	2

CY = colostrum yield (L); IgG = immunoglobulin G concentration (g/L); IgTOT = total immunoglobulin concentration (g/L); IgG yield = immunoglobulin G yield (g); GO:MF = gene ontology molecular function; GO:CC = gene ontology cellular components; GO:BP = gene ontology biological processes (GO:BP); KEGG = Kyoto Encyclopedia of Genes and Genomes pathways

[a]Terms are ordered by significance within each trait; $P \leq 0.10$.
